# Synchronized affect in shared experiences strengthens social connection

**DOI:** 10.1038/s42003-023-05461-2

**Published:** 2023-10-28

**Authors:** Jin Hyun Cheong, Zainab Molani, Sushmita Sadhukha, Luke J. Chang

**Affiliations:** Department of Psychological and Brain Sciences, Dartmouth College, Hanover, NH USA

**Keywords:** Human behaviour, Psychology

## Abstract

People structure their days to experience events with others. We gather to eat meals, watch TV, and attend concerts together. What constitutes a shared experience and how does it manifest in dyadic behavior? The present study investigates how shared experiences—measured through emotional, motoric, physiological, and cognitive alignment—promote social bonding. We recorded the facial expressions and electrodermal activity (EDA) of participants as they watched four episodes of a TV show for a total of 4 h with another participant. Participants displayed temporally synchronized and spatially aligned emotional facial expressions and the degree of synchronization predicted the self-reported social connection ratings between viewing partners. We observed a similar pattern of results for dyadic physiological synchrony measured via EDA and their cognitive impressions of the characters. All four of these factors, temporal synchrony of positive facial expressions, spatial alignment of expressions, EDA synchrony, and character impression similarity, contributed to a latent factor of a shared experience that predicted social connection. Our findings suggest that the development of interpersonal affiliations in shared experiences emerges from shared affective experiences comprising synchronous processes and demonstrate that these complex interpersonal processes can be studied in a holistic and multi-modal framework leveraging naturalistic experimental designs.

## Abstract (web summary)

Participants jointly watching a television show exhibit temporal synchrony of facial expressions and electrodermal activity, implying social connection and informing the design of more naturalistic experiments investigating social bonding.

## Introduction

People value shared experiences^1^. Experiences in large groups such as religious ceremonies, music concerts, and sporting events often involve synchronization in movements, vocalizations, feelings, and thoughts with others, which has been thought to strengthen group cohesion^2–6^. These amplified feelings of connection following a shared experience can also occur in everyday settings. For example, simply watching an emotional film rather than a neutral documentary in a crowd can foster feelings of connection with the audience^7^. However, we know very little about *how* the various interpersonal synchronous processes that occur in these social contexts contribute to the feeling that an experience is shared between individuals and how these experiences facilitate social connection.

Frequently observed group behaviors such as synchronous singing during concerts or simultaneous reactions during a movie have led to a common assumption that co-experiencing the same external stimuli elicits largely similar feelings and interpretations across people. However, just as people do not all enjoy the same scenes or like the same characters in a movie, individuals’ thoughts and feelings can be highly idiosyncratic even when observing the same event^8^ which may reflect differences in people’s attention, goals, and preferences^9–11^. These variations in endogenous signals can impact cognitive appraisals of unfolding events and the generation of affective meaning^9,12,13^. Consequently, individuals’ physiological arousal^4,14^, neural responses^10,15,16^, and facial expressions^17,18^ may converge and diverge throughout the course of an experience reflecting dynamically shifting alignments of thoughts and feelings^4,19,20^.

Synchronous shared experiences may be reflected in moment-to-moment temporal dynamics of facial expressions^21^. Most facial expression research, however, has traditionally focused on comparing summary statistics across experimental conditions such as average amplitudes or frequencies of an expression^22–25^. For example, counting the frequency of positive and negative facial expressions when watching a video revealed that roommates’ and dating partners’ responses to short video clips appear to become more similar over time^26^. However, this approach of measuring convergence does not directly test if individuals displayed equivalent positive and negative expressions at precisely the same moments in time. Alternatively, convergence measured this way could also arise as a result of emotional contagion in which one person’s emotional state directly influences another’s subsequent emotional state^27–33^. Therefore, examining moment-to-moment fluctuations in emotional facial expressions can provide a more nuanced understanding of how and when individuals truly “click” during shared experiences.

Individuals may also become more aligned in how they express certain emotions through different configurations of facial muscle movements during a shared experience. Though emotion research has typically focused on a limited number of canonical expressions^34^, recent efforts to uncover natural variations of emotional facial expressions in the wild and across cultures have highlighted considerable diversity in the specific spatial configuration of muscles that comprise a facial expression^35–38^. For example, subtle variations in smiles can convey vastly different meanings including affiliation, reward, or dominance to an observer^39,40^. Within an interaction, convergence on a shared ontology of communication signals (e.g., facial expressions) may improve the ease of communication and degree of social connection^41^. This can be observed in “chameleon” effects, in which individuals mutually adapt to each other’s behaviors during an interaction^42–52^. Greater degrees of behavioral similarity (e.g., smiling, face touching, and leg shaking) between individuals have been found to yield smoother and more enjoyable interactions^42,45^ although these findings may not be consistent across contexts^53,54^.

One challenge in recording and analyzing emotional facial expressions is that they can be voluntarily controlled by participants. Depending on the individual’s goal and environment, smiles and other expressions can be suppressed, feigned, or exaggerated. However, physiological responses to emotional events can be more difficult to control or suppress^55^ and have been found to be reliably elicited in response to emotional experiences^56^. For example, electrodermal activity (EDA), which reflects changes in the electrical characteristics of the skin, is thought to be a marker of autonomic arousal arising from the sympathetic nervous system^56–58^ and has been found to synchronize between family members going through painful rituals^4^, friends having an emotional conversation^59^, and patients and therapists in psychotherapy interviews^60^. These findings suggest that the synchrony of EDA may also be a reliable indicator of shared experiences and a contributor to developing stronger social connections.

**Fig. 1:**
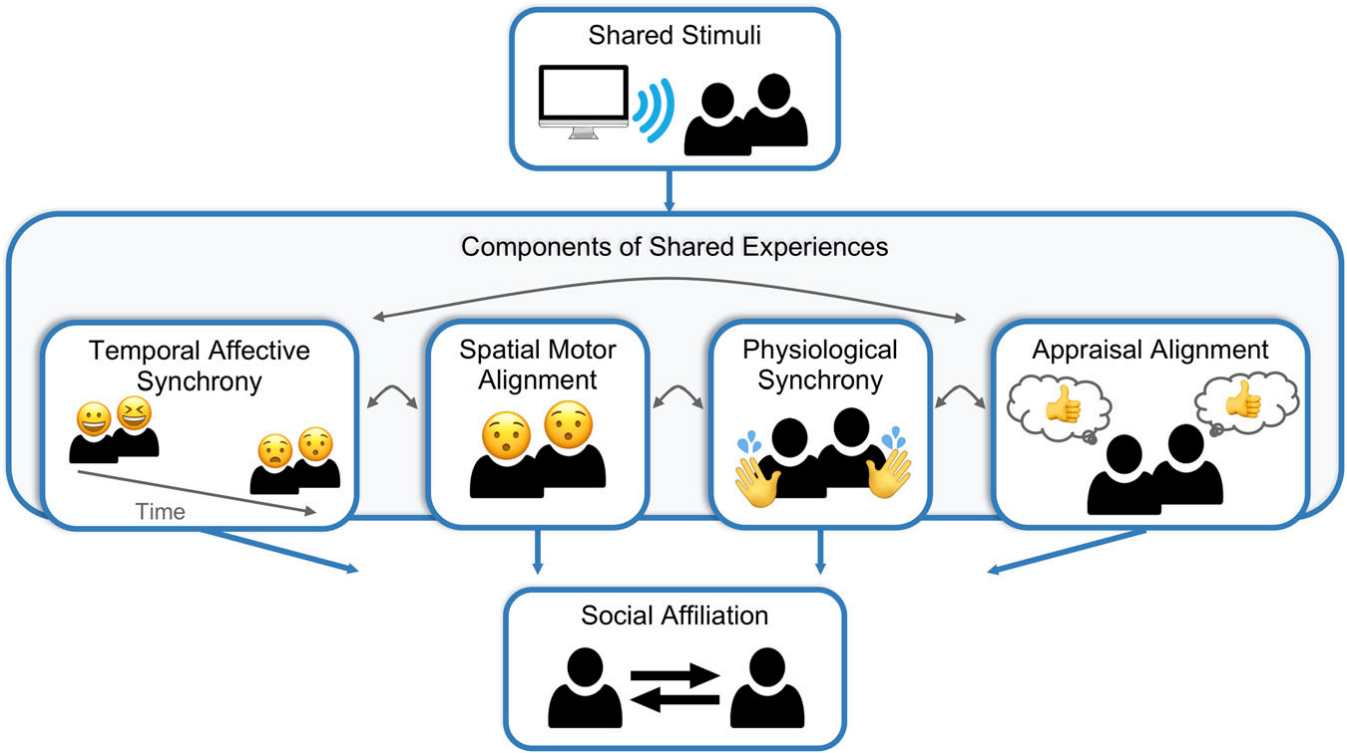
Components of shared experiences. Simultaneously experiencing a stimulus between individuals can lead to affective experiences synchronized in time (temporal affective synchrony), similar patterns of facial muscle activations in how emotion is displayed (spatial motor alignment), physiological synchrony, and similar cognitive appraisals of the experience (appraisal alignment) that help facilitate the development of interpersonal affiliation.

**Fig. 2:**
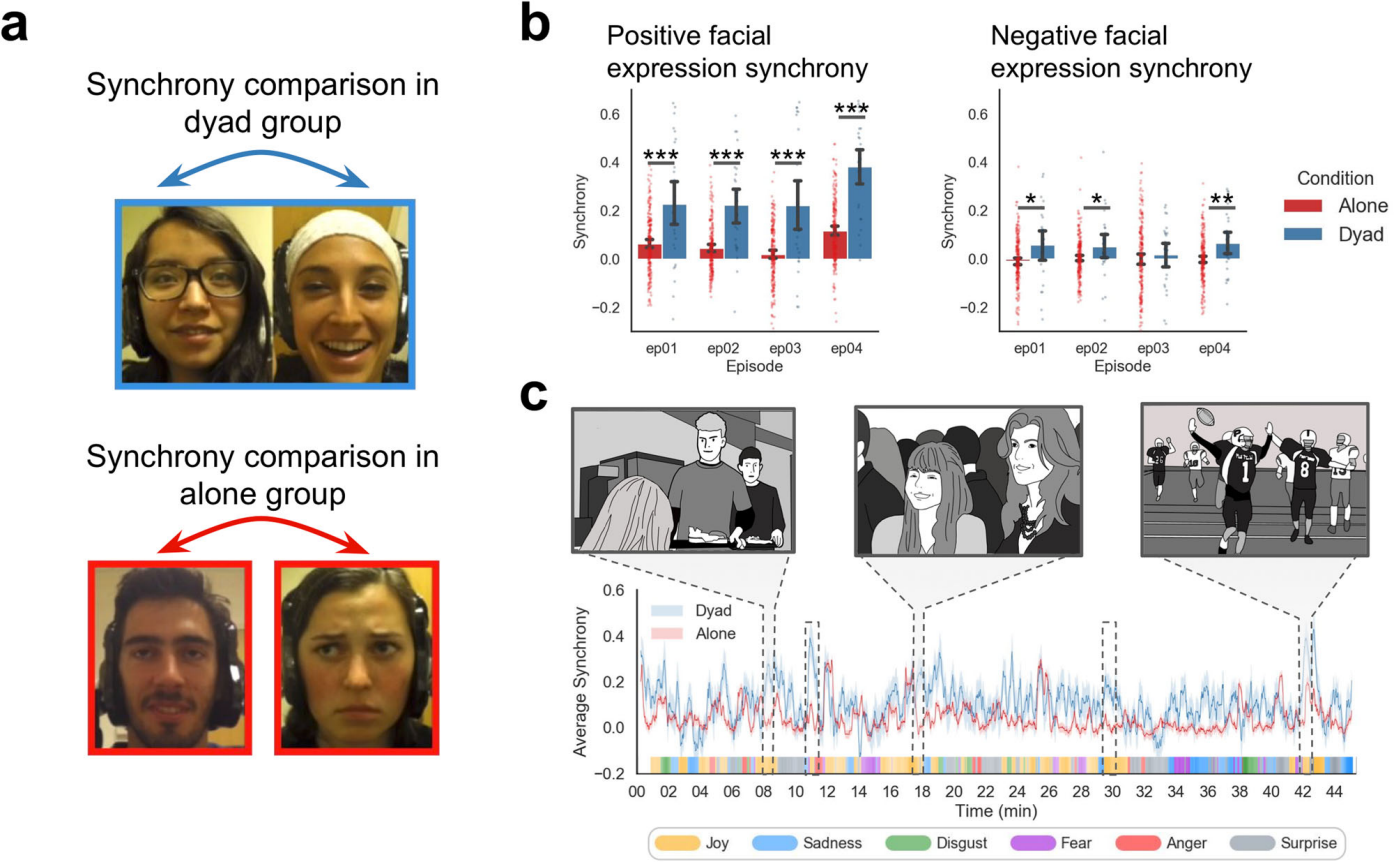
Temporal facial expression synchrony. **a** Examples of participant facial expressions. Synchrony in the alone group was computed between all pairwise participants who watched individually whereas synchrony in the dyad group was computed only between the pairs of participants who watched physically together sitting next to each other. Images are displayed with written consent from the participants. **b** Synchrony of positive facial expressions while watching episodes between conditions in each episode. Significance of group differences was tested with a two-sample permutation test (error bars indicate 95% confidence intervals *p < 0.05, **p < 0.01, ***p < 0.001). **c** Dynamic facial expression synchrony for the dyad and alone group in episode 1. Dotted boxes indicate clusters at which dyad participants synchronized more than alone participants. Select depictions for the significant clusters are shown above. These moments correspond to scenes with high joy ratings rated by *N* = 188 independent observers and are indicated by orange color bars along the *x*-axis (shaded areas indicate standard error of the mean). Colored ticks along the horizontal axis indicate the most representative emotion at that time from online crowdsourced self-reported emotion ratings (see Supplementary Methods). Original illustrations of highlighted scenes depicted from the *Friday Night Lights* television show were created by Dr. Stephanie Lee and shared with permission.

In this study, we examined how the alignment of facial expression behavior, electrodermal activity, and thoughts emerge during a shared experience and how the experience contributes to the development of social connection (Fig. 1). We used a naturalistic paradigm, in which participants (*N* = 86) watched four episodes of a character-driven television drama, *Friday Night Lights*, for approximately 4 h over two viewing sessions. Participants either watched the show alone or in a dyad with another previously unacquainted participant seated side-by-side with each other in the same room (Fig. 2a). During the show, we continuously recorded participants’ faces using head-mounted cameras^61^ to probe moment-to-moment displays of facial muscle movements. We used a pre-trained convolutional neural network^62^ to convert each video frame into predicted emotions and action units, which reflect the firing intensity of different groups of facial muscles^18^. We also measured the physiological arousal response of participants while viewing the show by recording their EDA^56–58^. After each episode, participants independently rated their impressions of the characters (e.g., How much do you like this character? How much do they remind you of someone you know?) and dyads rated how connected they felt to their viewing partner. We hypothesized that participants watching videos in dyads would exhibit greater synchronization of emotional facial expressions and EDA and that the degree of these synchronizations would correlate with stronger reports of feeling connected to their viewing partners. We also predicted that participants with more similar character impressions would report feeling more connected to one another. Finally, we disentangled how these synchronous processes that concurrently arise during a shared activity contributed to the feeling that they were not merely attending to the same stimuli but were actually sharing similar experiences in time that ultimately helped form social bonds.

## Results

### Temporal synchrony of affective facial expressions

First, we evaluated if participants watching the show in a social context exhibited greater synchronization of facial expressions compared to those who watched alone. We specifically examined the temporal dynamics of the predicted intensity emotions from the facial expression behavior aggregated into positive and negative valence at each frame of the video. Next, we computed the temporal alignment between participants by calculating the Pearson correlation between pairs of participants’ facial expression trajectories for positive and negative emotions separately for each group per episode. Significance was tested using two different types of nonparametric resampling approaches^63^—subject-wise bootstrapping^64^ and generating surrogate data by circularly shifting the time series^65^. In episode 1, we found significant synchronization in positive facial expressions (i.e., joy facial expressions) for both the dyad (Pearson *r*_Episode1_ = 0.23, SD = 0.25, *p* < 0.001) and the alone group (*r*_Episode1_ = 0.06, SD = 0.13, *p* < 0.001), with comparable significant results in all subsequent episodes (Fig. 2b; Supplementary Table S1). Average synchrony also increased over time for both the dyad (*β* = 0.05, t(28) = 4.68, *p* < 0.001) and the alone group (*β* = 0.01, t(14) = 2.32, *p* = 0.04).

Across all episodes, positive facial expression synchrony in dyads was significantly higher than that of participants who watched alone with an average difference of *β* = 0.20, *t*(31) = 5.21, *p* < 0.001, and this difference increased over subsequent episodes, *β* = 0.03, *t*(34) = 3.20, *p* = 0.003. To test if the increased synchrony was specific to the dyads who watched together or a general phenomenon of social viewing, we compared the synchrony of paired dyads to random combinations of non-paired dyad participants. These are random pseudo-dyads created from participants of the dyad group who did *not* physically watch the episodes together. Across episodes, we found that paired dyads exhibited greater synchronization compared to non-paired dyads with an average correlation difference of *β* = 0.13, *t*(28) = 6.81 *p* < 0.001, and non-paired dyads synchronized more than participants that watched alone with a difference of *β* = 0.07, t(74) = *p* < 0.001 (Supplementary Fig. S1). For negative facial expressions, represented by the maximal value across negative emotions such as anger, fear, disgust, sadness, and contempt^66^, we did not observe a significant main effect of synchronization. However, dyads did synchronize more than the alone group in specific episodes (episode 1: *r* = 0.07, *p* = 0.01; episode 2: *r* = 0.05, *p* = 0.047; episode 3: *r* = 0.02, *p* = 0.44; and episode 4: *r* = 0.07, *p* = 0.0013) (Fig. 2b). As the synchrony was minimal for negative facial expressions, the remainder of our analyses focused on positive facial expressions unless otherwise stated.

Next, we examined whether positive facial expression synchrony was driven by specific events during the show for the first episode. To test this we used a 30-s sliding window correlation to compute the dynamic time-varying positive facial expression synchrony at every second of the show (Fig. 2c). These trajectories were then compared with self-reported emotion ratings from participants recruited on Amazon Mechanical Turk watching episode 1 (*N* = 182) collected from a previous study^9^. Overall, we found that the dynamic positive facial expression synchrony in dyads positively correlated with average subjective feelings of joy (*r* = 0.24, *p* < 0.001) and negatively with feelings of sadness (*r* = −0.12, *p* < 0.001). We then located specific moments in the show when dyads displayed greater synchrony than the alone group using a cluster-based permutation test^67^. This analysis identified five short temporally contiguous scenes in which dyads synchronized more than the alone group in Episode 1 (see Supplementary Fig. S2 for other episodes) including scenes depicting: (1) two male characters awkwardly approaching a female character (8th minute), (2) many characters socializing at a party (18th minute), and (3) an underdog character scoring a game-winning touchdown (42nd minute). These scenes appeared to evoke largely “feel-good” or humorous feelings, as average joy ratings were significantly higher than other crowdsourced emotion ratings, *β* = 12.14, *t*(24) = 4.08, *p* < 0.001 (Supplementary Fig. S3).

In summary, co-viewing led to significant temporal synchrony of positive facial expressions during shared experiences beyond synchrony levels that are purely stimulus-driven. All viewers tended to synchronize positive facial expressions during amusing scenes, but this effect was stronger when participants watched with another person in the dyad condition. These stronger synchronizations were observed in both long (45-min episodes) and short timescales (30-s sliding windows) and corresponded to positive sentiments of the show based on crowdsourced emotion ratings.

### Temporal affective synchrony and self-reported social connection

**Fig. 3:**
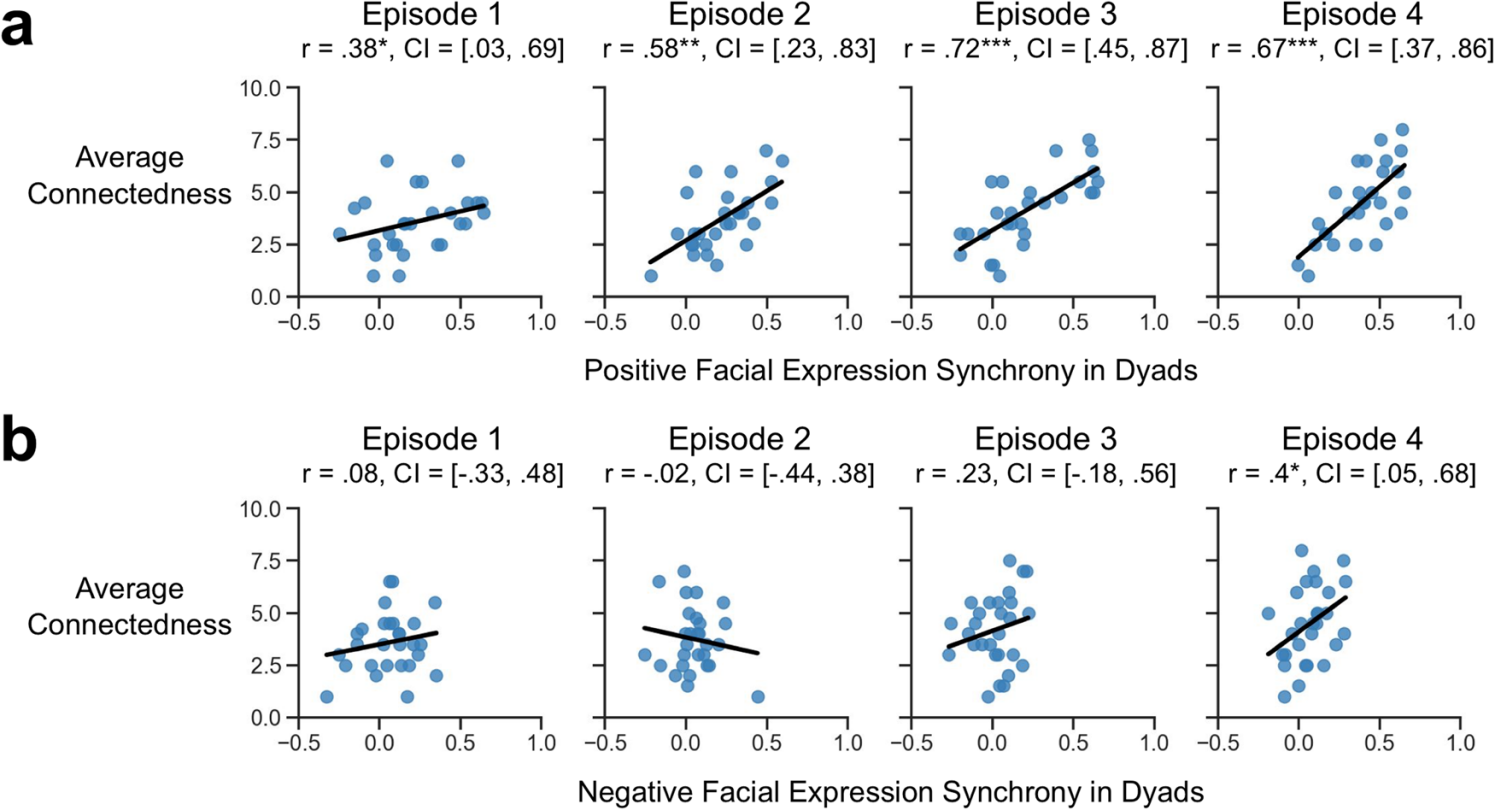
Correlation between connectedness and facial expression synchrony in each episode. **a** Synchrony of positive facial expressions and average connectedness ratings after each episode. **b** Synchrony of negative facial expressions and average connectedness ratings after each episode. CI indicates subject-wise bootstrapped 95% confidence interval. *p < 0.05, **p < 0.01, ***p < 0.001.

Next, we examined the relationship between global temporal affective synchrony and the development of affiliation. After each episode, participants were asked, “How much do you feel connected to the other participant?”. We used a linear mixed-effects regression to predict the average of how connected each participant reported feeling to their viewing partner and found that temporal synchrony of positive facial expressions in dyads significantly contributed to the prediction across all four episodes, *β* = 1.04, *t*(88) = 3.36, *p* = 0.001 (Fig. 3a). We also observed a significant effect of episode number, *β* = 0.25, *t*(81) = 5.96, *p* < 0.001, indicating that average connection ratings generally increased over time. An interaction between episode number and positive facial expression synchrony, *β* = 0.38, *t*(81) = 2.15, *p* = 0.034, revealed that the relationship between positive emotional synchrony and social connection strengthened over time. Negative expression synchrony was not significantly related to connectedness except in the last episode (Fig. 3b).

In order to assess how the synchrony of other canonical emotional facial expressions contributed to developing interpersonal connections, we trained a penalized regression model to predict self-reported connection ratings using the temporal synchrony of different emotional facial expressions (i.e., joy, anger, fear, disgust, surprise, and sadness). Using leave-one-dyad-out cross-validation, we were able to accurately predict connectedness in new dyads, *r* = 0.51. We used a linear mixed-effects regression to evaluate the independent contribution of each type of expression while controlling for time effects and found that only the synchrony of joy facial expression significantly predicted feelings of social connection, *β* = 0.78, t(80) = 2.16, *p* = 0.034. This indicates that synchrony of positive facial expressions was the most reliable contributor in explaining variations in connection, although this may be in part due to the nature of the stimuli that is mostly driven by positive events based on the crowdsourced emotion ratings for episode 1 (Supplementary Fig. S4).

### Spatial alignment of facial expressions and self-reported social connection

Previous analyses examined the synchronization of canonical expressions of specific emotions, but it is possible that individuals have subtle nuances in *how* they smile or frown and that shared experiences might facilitate convergence to a dyad-specific facial expression ontology^41^. To investigate this question, we employed a data-driven approach that allows the estimation of subject-specific facial expressions representing common emotional trajectories across subjects. We compared the spatial similarities in these idiosyncratic facial expressions between dyads to assess if a greater alignment was associated with increased feelings of social connection.

To estimate idiographic facial expression models representing similar affective response trajectories across participants, we fit a reduced-rank two-dimensional shared response model^68^ to the 20 facial action unit muscle movements time series^69^. This model estimates common latent temporal components across participants but allows each person to have a unique spatial configuration for a facial expression that is represented by the subject-specific weight matrix that projects participant data into the common latent space shared across participants.

**Fig. 4:**
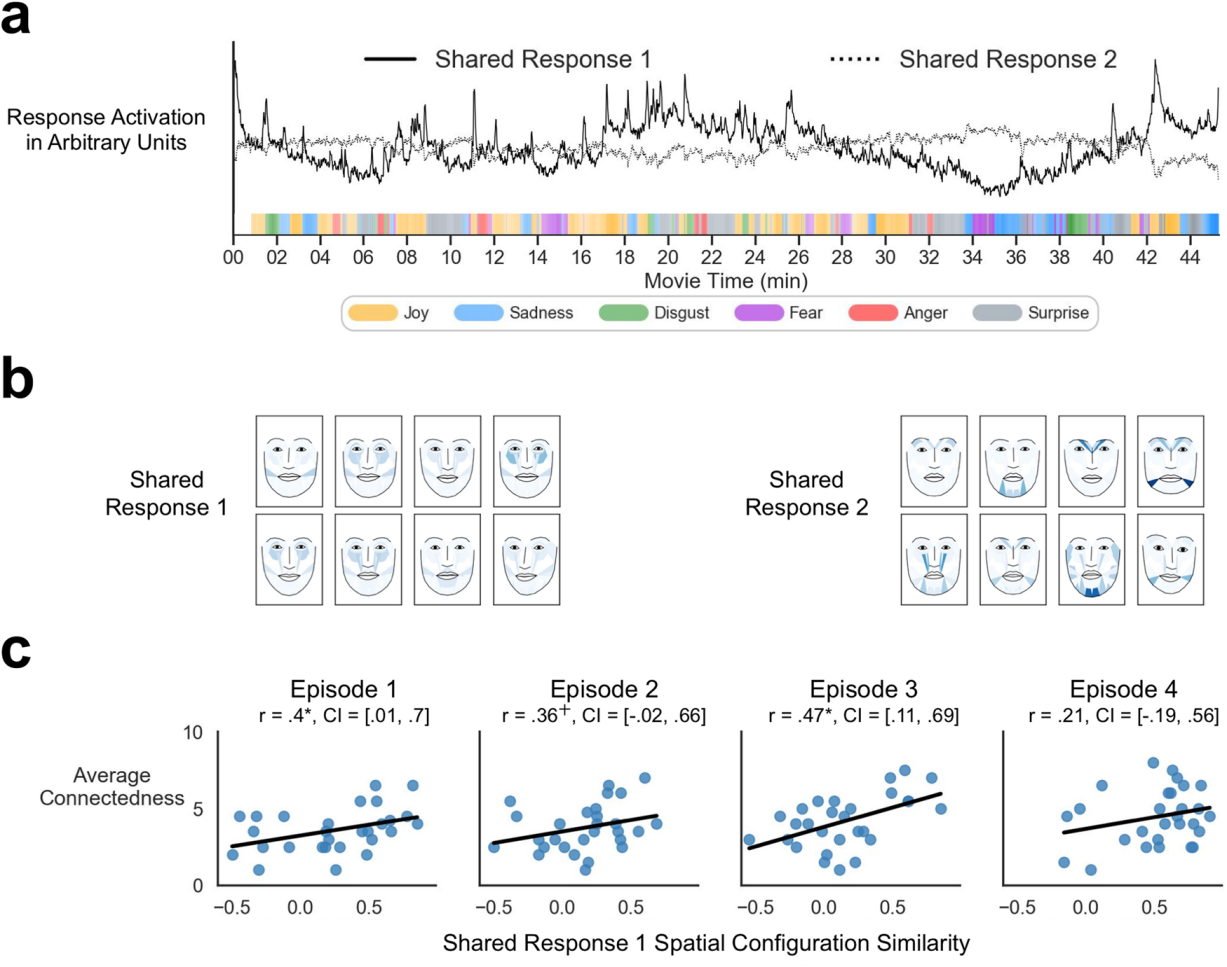
Spatial configuration synchrony results. **a** Estimated average shared response trajectories. The trajectory of the first shared response (Shared Response 1; solid line) included peaks at scenes when crowdsourced joy ratings (orange) were high, such as when the characters were joking around or when a football team won the match. The trajectory of the second shared response (Shared Response 2; dotted line) included peaks at scenes when crowdsourced fear ratings (purple) were high, such as when the coach was warned about the social consequences of losing a football match or when the main character gets injured during a football match. Shared response trajectories were linearly detrended and standardized for visualization. Colored ticks along the horizontal axis indicate the most representative emotion at that time from online crowdsourced self-reported emotion ratings. **b** Examples of participant-specific spatial patterns of action units activated for each shared response. Note the variability in expressions despite their occurrences are aligned to the shared response trajectories shown in Fig. 4a. These values have been exponentiated for display purposes. Faces are generated using open-source py-feat software which allows facial expressions to be displayed without revealing participants identity^98^. **c** Shared Response 1 spatial configuration pattern similarity is associated with connectedness ratings in dyads. CI indicates subject-wise bootstrapped 95% confidence interval. ^十^*p* < 0.1, *p < 0.05, **p < 0.01, ***p < 0.001.

This data-driven approach was able to successfully capture subjective facial expression variations representing shared affective trajectories. The first shared response correlated with the crowdsourced joy ratings (*r* = 0.42, *p* < 0.001) and peaked at positive scenes such as when the characters were winning the football match (42nd minute) or when they were joking around (17th minute; Fig. 4a). Interestingly, even though we placed no constraints on how to combine the action units to represent a shared response, we were able to extract participant-specific variations in spatial configurations of facial expressions (Fig. 4b, left panel) that resembled positive emotion expressions with activations in action units 6 (cheek raiser), 10 (upper lip raiser), 12 (lip corner puller), and 14 (dimpler).

The second shared response correlated with crowdsourced fear ratings (*r* = 0.29, *p* < 0.001) and sadness ratings (*r* = 0.23, *p* < 0.001) and peaked at negative or serious scenes, such as when the football coach was warned about the potential consequences of losing a match (14th minute) and when one of the main characters sustained a serious injury (34th minute; Fig. 4a). We observed diverse, subject-specific spatial configurations of facial expressions for this shared response (Fig. 4b, right panel) that resembled facial expressions associated with negative feelings including activations of action units 4 (brow lowerer), 5 (upper lid raiser), and 15 (lip corner depressor).

Next, we tested if viewing partners with more similar spatial patterns of facial expressions developed stronger social connections. Participants sat physically next to each other as they viewed the episodes allowing them to freely see each other’s expressions. To evaluate the relationship between the spatial alignment of facial expressions and social connection, we computed the spatial similarity of subject-specific facial expression patterns for each dyad and correlated these synchrony measures with the dyad’s average self-reported feelings of social connection. Connection ratings were positively correlated with spatial facial expression synchrony of the first shared response (Fig. 4c) in episodes 1 (*r* = 0.4, *p* = 0.036, 95% subject-wise bootstrapped confidence interval CI = [0.01, 0.7]), episode 2 (*r* = 0.36, *p* = 0.062, CI = [−0.02, 0.66]), and episode 3 (*r* = 0.47, *p* = 0.011, CI = [0.11, 0.69]) but not in episode 4 (*r* = 0.21, *p* = 0.292, CI = [−0.19,0.56]), indicating that similarities in how positive emotions are expressed may contribute to stronger social affiliation and feelings of a shared experience. In contrast, connection ratings were not significantly correlated with spatial synchrony of the second shared response in any of the episodes.

### Synchrony of electrodermal activity and self-reported social connection

**Fig. 5:**
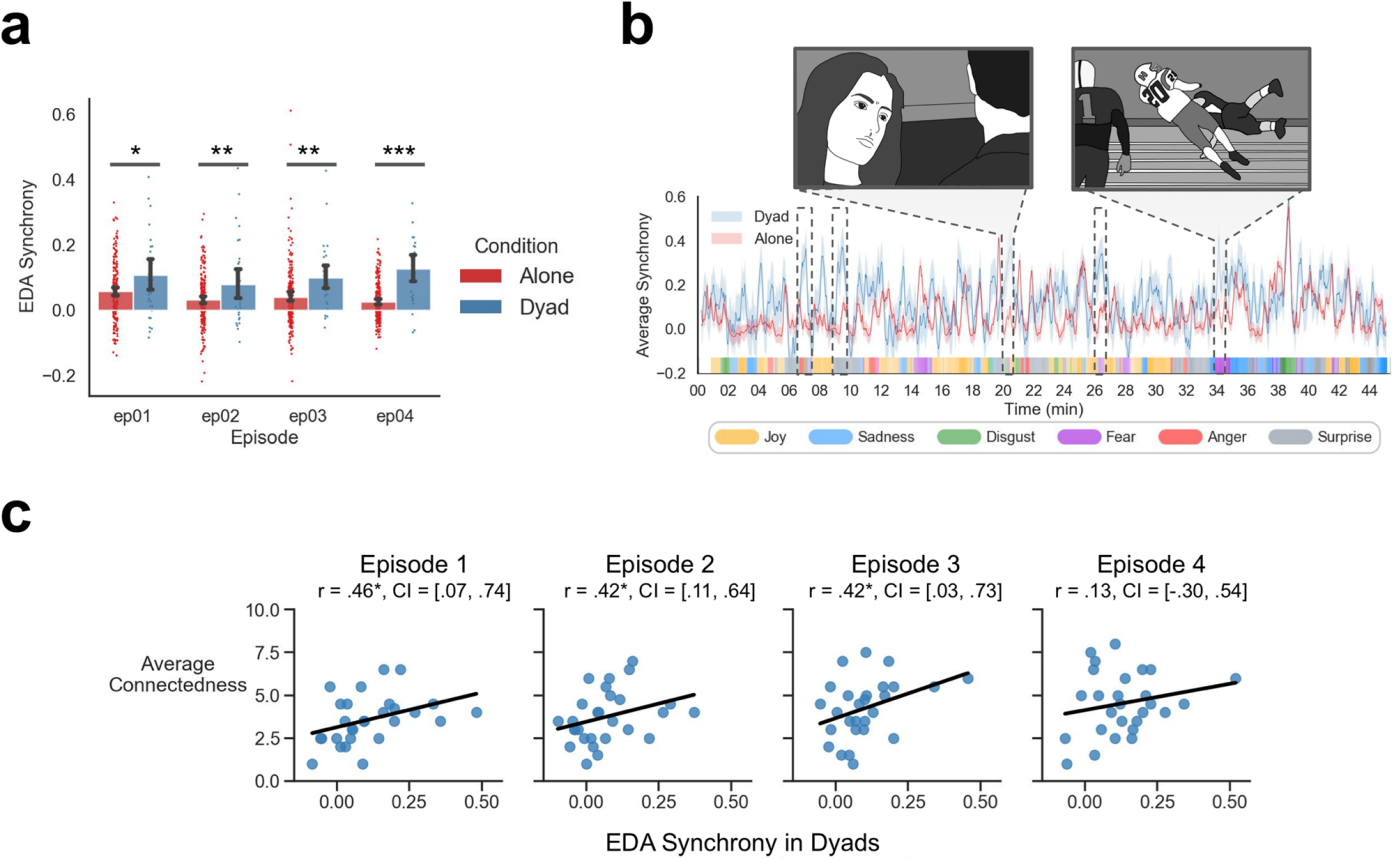
EDA synchrony. **a** Average global EDA synchrony in the dyad and alone groups across each 45-min episode. **b** Dynamic EDA synchrony of dyad and alone groups. Dashed boxes show moments when the EDA synchrony of dyads was significantly greater than the EDA synchrony of alone participants. The dominant crowdsourced emotions at these scenes included fear, anger, and surprise. A sample image from select scenes is depicted in shaded boxes showing football players smashing into each other (7th minute), couples having a verbal argument (20th minute), and a football player getting seriously injured (34th minute). Shaded areas indicate standard error of the mean. Colored ticks along the horizontal axis indicate the most representative emotion at that time from online crowdsourced self-reported emotion ratings. **c** Effect of EDA synchrony on average self-reported connectedness ratings for dyads. Brackets show subject-wise bootstrapped 95% confidence intervals on the correlation estimates. *p < 0.05, **p < 0.01, ***p < 0.001. Original illustrations of highlighted scenes depicted from the *Friday Night Lights* television show were created by Dr. Stephanie Lee and shared with permission.

Participants’ sympathetic autonomic nervous system activity was measured via EDA to complement facial expression behavior. We observed a significant intersubject EDA synchrony for both the dyad, *r* = 0.11, SD = 0.13, *p* < 0.01, and alone groups, *r* = 0.06, SD = 0.09, *p* < 0.001, tested through subject-wise bootstrapping against the null correlation and generating surrogate data by randomly circle-shifting the time series (Supplementary Table S1). Comparing the level of EDA synchrony between the two groups revealed that dyads exhibited greater global EDA synchrony compared to the alone group, β = 0.06, t(20) = 4.92, *p* < 0.001 (Fig. 5a) and this group difference showed an increasing trend over time, β = 0.02, t(26) = 2.00, *p* = 0.056.

We further investigated the type of scenes that elicited dynamic EDA synchrony using a cluster-based permutation test^67^ to identify temporal epochs where the dyads synchronized more than those who watched alone. In episode 1, dyads synchronized significantly more than the alone participants in five scenes outlined by dashed boxes in Fig. 5b. The dominant emotion in these scenes from the crowdsourced ratings included: fear, anger, and surprise. This is consistent with the narrative events that depicted negative and highly arousing events such as football players smashing into each other (7th minute), couples having a verbal argument (20th minute), and a star football player becoming seriously injured (34th minute) (Fig. 5b). We sought to more precisely characterize this signal by identifying the crowdsourced emotion ratings that best predicted the temporal dynamics of EDA synchrony using a mixed-effects regression. We found that dynamic EDA synchrony in episode 1 was best explained by negative emotion ratings such as fear β = 0.16, t(2623) = 8.68, *p* < 0.001 and disgust β = 0.12, t(2623) = 7.54, *p* < 0.001, and somewhat by surprise β = 0.05, t(2623) = 3.05, *p* < 0.001 and joy β = 0.05, t(2623) = 3.87, *p* < 0.001. We observed similar relationships with negative facial expressions across all four episodes. EDA synchrony was significantly predicted by average facial expressions of fear β = 0.08, t(10362) = 7.79, *p* < 0.001, disgust β = 0.14, t(10362) = 10.84, *p* < 0.001, and surprise β = 0.03, t(10362) = 3.60, *p* < 0.001, while sadness β = −0.05, t(10362) = −5.21, *p* < 0.001 and joy β = 0.03, t(10362) = −6.20, *p* < 0.001 facial expressions predicted decreased EDA synchrony.

We also explored the relationship between global EDA synchrony with self-reported social connection ratings. Consistent with the previous analyses, we found that the degree of EDA synchrony in dyads predicted how connected participants reported feeling to one another, β = 1.75, t(81) = 3.77, *p* < 0.001 (Fig. 5c). This effect was stronger in episodes 1, 2, and 3, but did not significantly change with time, β = −0.01, t(80) = −0.04, *p* = 0.96. Together, these results indicate that shared negative affective experiences also contributed to increased feelings of social connection. However, unlike positively valenced experiences, participants may not be consistently expressing these negative feelings via their facial expression behavior. This inconsistency in physiological and behavioral indicators suggests that cultural norms may be influencing how participants socially display negative feelings in this particular social context.

### Similarity of character impressions and self-reported social connection

**Fig. 6:**
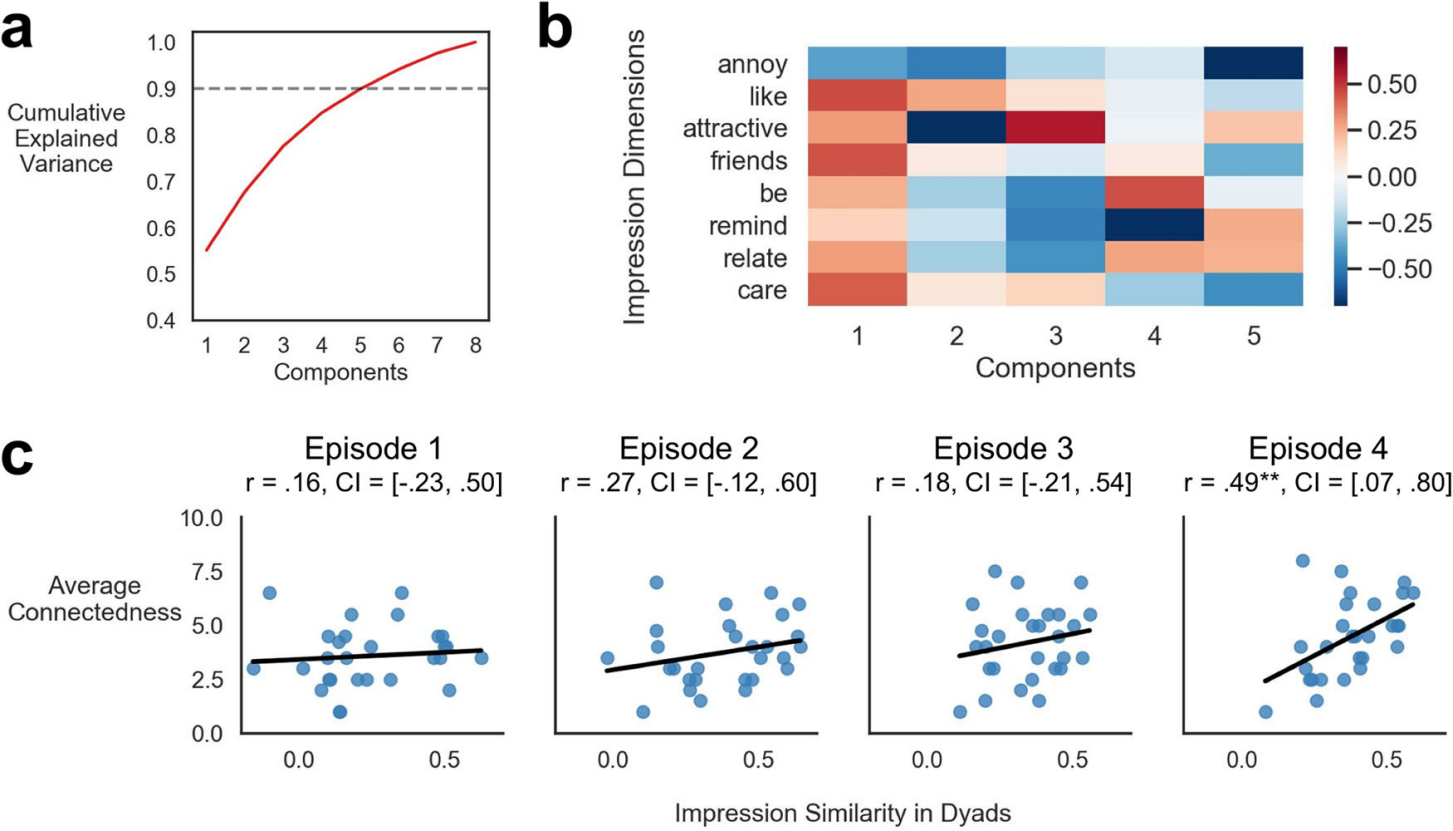
Similarity of character impressions. **a** Cumulative explained variance of principal components across impression ratings. The dotted line indicates 90% cumulative explained variance. **b** Component loadings on each impression dimension. **c** Correlation between impression similarity and dyad average connection ratings for each episode. CI indicates subject-wise bootstrapped 95% confidence interval. ^十^*p* < 0.1, *p < 0.05, **p < 0.01, ***p < 0.001.

Next, we examined if dyads with similar impressions of the characters reported higher levels of connectedness to their viewing partner. To account for potential overlap and redundancies in the collected impression ratings (e.g., ratings of liking and wanting to be friends with a character were correlated at *r* = 0.72), we reduced the dimensionality of the ratings using a Principal Components Analysis while preserving approximately 90% of the explained variance with 5 components (Fig. 6a). The first component which accounted for 55% of the variance represented a generally positive sentiment towards characters loading on questions related to liking and how much participants wanted to be friends with characters (Fig. 6b). The second component also loaded on liking ratings but with loadings in the opposite direction for attractiveness suggesting a dimension of a positive impression without physical attraction. In contrast, the third component strongly reflected the attractiveness of characters. The fourth component represented an impression of how much the participants wanted to be a certain character and a negative loading on how the characters reminded the participants of someone they knew. The fifth component which accounted for the least amount of variance (5%) represented negative loadings on the annoyance of characters but with positive loadings on judgements of character attractiveness, and also how much participants related to the characters and reminded them of someone they know in real life.

Using these latent impression dimensions as an embedding space, we computed the intersubject similarity of impressions^10,11,15^ for each character and averaged the similarity of characters for each dyad. Across episodes, dyads with higher average impression similarity across characters also showed greater connection ratings, *β* = 1.01, *t*(85) = 2.40, *p* = 0.018, and this association was most pronounced in the last episode, *r* = 0.49, *p* = 0.009 (Fig. 6c). In addition, impression similarity among dyads increased after each episode, *β* = 0.03, *t*(83) = 3.05, *p* = 0.003.

### Shared experience as a latent process

**Fig. 7:**
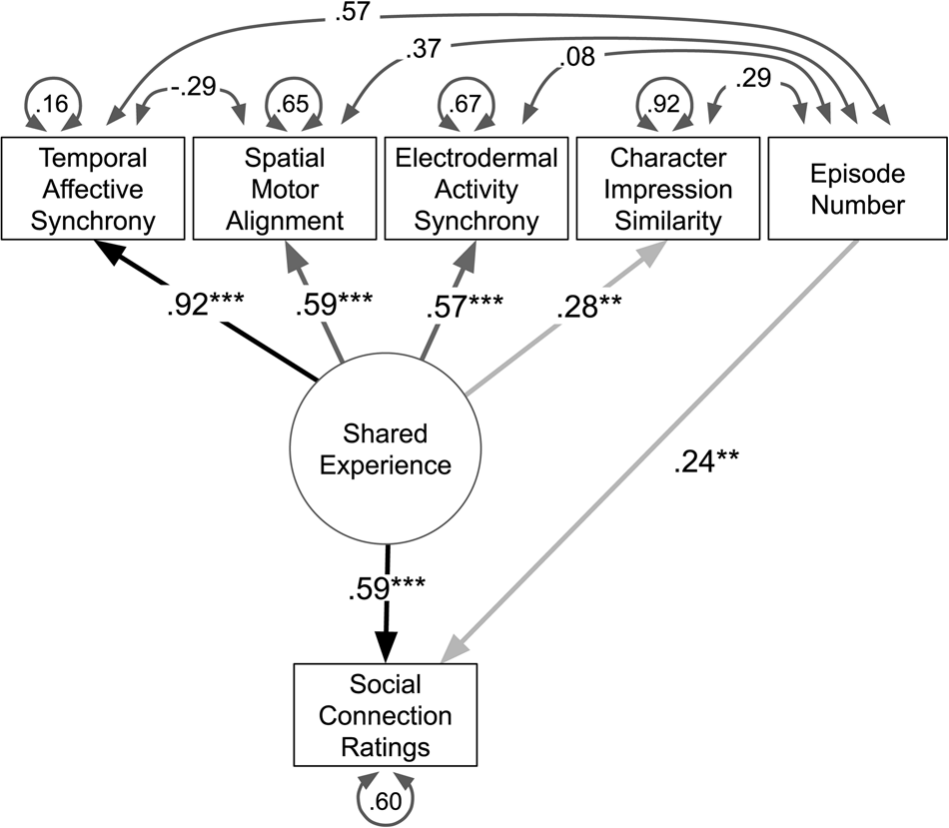
Path diagram of relationships between synchrony measures, shared experience, and connection ratings estimated using structural equation modeling. Here we plot the path diagram of our structural equation model. Measured variables are depicted with a square and include temporal affective synchrony, spatial motor similarity of facial expressions, EDA synchrony, similarities in character impression ratings, and social connection ratings. Shared experience is modeled as a latent variable and depicted with a circle. Direct relationships are visualized with straight lines with a single arrow. Covariance between variables is visualized using curved lines with two arrows. The disturbance or unexplained residual error for each measured variable is depicted using circular arrows. Numerical values along the paths indicate standardized contributions. Overall, we find that temporal affective synchrony most strongly loads on the latent shared experience experience variable, followed by spatial motor synchrony and character impression similarity. Shared experiences, in turn, predict social connection controlling for the amount of time spent by participants together during the experiment (episode number). *p < 0.05, **p < 0.01, ***p < 0.001.

The previous results demonstrate that synchronization of facial expressions in time and space, synchrony of EDA, and similar appraisals of characters are all associated with developing feelings of connection when viewing a show with another person. An important question is whether these different measurements reflect independent constructs or are manifestations of a common latent shared experience. To test this question, we used structural equation modeling to estimate a latent factor model using synchronization and similarity measures across all of our data^70,71^. Our model predicted connection ratings using episode number to indicate time, and a shared experience latent factor measured by manifestations of temporal affective synchrony, spatial affective synchrony, EDA synchrony, and impression synchrony (Fig. 7). As hypothesized, temporal synchrony, spatial alignment, EDA synchrony, and impression similarity were all significant components of the shared experience (Fig. 7; Supplementary Table S2) and higher levels of shared experience significantly predicted higher social connection ratings (standardized variance = 0.59, *p* < 0.001) conditioning on the amount of time spent together (standardized variance = 0.24, *p* < 0.01).

## Discussion

In this study, we investigated how shared affective experiences can facilitate social connection. Using a naturalistic viewing paradigm in which participants spent approximately 4 h watching a character-driven television drama with a viewing partner, we found evidence that greater synchronization of affective experiences leads to increased feelings of social connection. This effect was observed across multiple measurements of the affective experience. We used computer vision algorithms to extract facial muscle movements from recorded videos of participant faces to objectively and unobtrusively measure facial expression behavior unfolding over time, and found that temporal synchronization of positive facial expressions and the spatial alignment of these expressions both contributed to increased feelings of social connection. In addition, we found that synchronization of sympathetic arousal responses measured by continuous recordings of electrodermal activity (EDA) predicted reports of social connection. Finally, we found that alignment in cognitive impressions of the characters also led to increased feelings of social connection. Using structural equation modeling, we demonstrated that each of these four distinct manifestations of the shared experience independently contributed to feelings of social connection within a dyad, even when controlling for the amount of time spent together. Together, this work demonstrates how acquiring unobtrusive recordings of individuals interacting in a naturalistic context can provide a more comprehensive view of the multidimensional and dynamic processes underlying social interactions^72^.

Why do individuals feel more connected after a shared experience? It has long been speculated that individuals form tighter social bonds after sharing a meal, watching a movie, or attending a concert or sporting event together and that they value and prefer shared experiences over solo experiences^1^. People may seek shared experiences because they provide opportunities for comparing their views of reality with others’^73^ through which individuals can satisfy epistemic motives such as wanting to establish socially approved beliefs and worldviews^74^ or relational motives such as the basic need for a sense of belonging^75^. This is consistent with work demonstrating that people have a preference for homophily, which leads individuals to seek out interactions with others who share similar backgrounds, beliefs, and behaviors^20,76^. Our findings point to potential mechanisms that support this process. Synchronous facial expressions may act as social signals that communicate the presence and development of shared feelings and thoughts about unfolding events. This is congruent with growing evidence that synchronization of neural response patterns can provide direct evidence of shared interpretations^77,78^, memories^79^, and segmentations^80^ of narrative events with respect to varying goals^81^ and feelings^9,15,16,82^. For example, laughing at the same time might signal that a dyad interpreted an event in the same way and imply that the two participants share a similar view of the world or sense of humor. Consistent with this hypothesis, we found that participants exhibited greater synchronization of smiling behavior during scenes reported to be positively valenced when watching with another person than by themselves. Further, dyads that reported similar cognitive impressions of each character (e.g., liking, annoyance, attractiveness, etc) were more likely to feel connected with one another than dyads that deviated in their impressions. Together, these findings suggest that knowing that another person is thinking and feeling the same way as you is likely to increase feelings of closeness with that particular individual.

Our work provides new insight into the temporal scale and dynamics of shared experiences. We show that simply having individuals attend to and watch the same stimuli did not automatically foster feelings of social connection with one another. Rather, individuals had to be able to feel and display similar emotional reactions at the same moments in time to develop feelings of connection to one another. We found that the median offset in synchrony within dyads was only 850 ms across all four episodes (Supplementary Fig. S5). Though previous work has presented evidence that synchronization of affective responses is linked to shared experiences, very few studies have actually assessed temporal synchronization at a precise time scale. For example, prior work has found that social contexts can amplify the intensity and frequency of emotions^22,24,25^ and response patterns^26,83^ for a variety of affective experiences such as tasting a sweet or bitter chocolate^84^ or watching video clips^85,86^, but none of these studies have demonstrated that these shared experiences occurred at the same moments in time. Further, related phenomena such as emotion contagion^29,44^ or mimicry of bodily movements^45^ indicate that these social modulations can occur in temporal succession between individuals, which necessarily implies that these feelings are not phase-synchronized. Positive feedback loops of these feelings between individuals may provide a mechanism for the amplification of the intensity of the experience, but this might be a computationally distinct mechanism from the phase-synchronized processes observed in our data. Evaluating different models of these complex dynamics will be critical in future efforts to characterize the psychological and neurophysiological mechanisms that gave rise to these effects^87–90^.

This study raises additional questions regarding the importance of the *type* of affective experience in facilitating social connection. We find strong evidence that positive facial expressions occurring at primarily positive events are more likely to synchronize across individuals and predict feelings of social connection compared to facial displays of negative emotions. We did observe a small effect of synchrony of negative facial expressions, but this did not reliably relate to feelings of social connection. One possible explanation for these discrepancies is that our stimuli did not strongly elicit negative emotional experiences. However, we do not think this explanation is likely as the base rates of positive and negative feelings identified in our crowdsourced emotions in the first episode were fairly comparable across positive and negative feelings (Supplementary Fig. S4). Future work should consider exploring other film genres (e.g., thriller or horror) to see if these findings are stimuli specific. Another possibility is that our computer vision models used to identify facial expression behavior are systematically less accurate for negative emotions compared to positive emotions^91^. This is certainly a possibility that we unfortunately are unable to rule out in this study. Further, even if the facial expression models are equally accurate across emotions, there may be greater variation in how negative events are experienced by participants relative to positive events. This is also consistent with a general bias in emotion research to focus on many different types of negative emotions (e.g., anger, sadness, disgust, etc) but very few positive emotions (e.g., joy). Finally, there may be strong cultural norms about how emotions are displayed in this particular social context. Facial expressions can be feigned, suppressed, and exaggerated, which may obfuscate signaling of internal feelings and thoughts^22,69,92^. Suppressing or hiding negative feelings in public (e.g., crying) is likely to reflect cultural norms that will vary across contexts (e.g., funerals, weddings) and upbringing^93,94^. In this study, participants are meeting a stranger for the first time and watching a video in close physical proximity during a laboratory experiment. It is possible that participants do not feel comfortable or deem it appropriate to display negative emotions in this context. We find some evidence supporting this potential interpretation. Unlike negative facial expressions, we did observe that EDA synchrony increased during negative scenes and somewhat correlated with the synchrony of negative facial expressions. This inconsistency in physiological and behavioral indicators suggests that cultural norms may be influencing how participants display negative feelings in this particular social context.

How generalizable are these findings to other social contexts? In this study, we were specifically interested in examining shared affective experiences in a passive viewing context, in which participants could naturally interact via verbal and non-verbal communication. Most dyads did not directly speak to each other very often (71% spoke less than 5 min across 4 h viewing sessions; median talking duration across episodes = 9.75 s; median number of utterances per episode was 5.75), but they undoubtedly communicated nonverbally throughout the viewing episodes. This paradigm is representative of many different everyday contexts (e.g., passively viewing movies, performances, sporting events, or religious ceremonies) and allows us to directly compare affective experiences in non-social contexts while minimizing the impact of talking on facial expressions and motion on physiological signals. However, there are many other social contexts that involve more direct verbal interactions (e.g., dyadic conversations or group discussions). We would not necessarily expect to see the same type of synchrony of affective experiences in more direct social exchanges as conversations require taking turns and necessarily force synchrony to be offset in phase. However, many communicative signals such as facial expressions, laughter, or physiological signals could still be occurring at the same moments in time. A promising avenue for future research is to assess the generalizability of these findings to other contexts with particular attention to the temporal scale and dynamics between individuals as they interact.

In summary, we find evidence for multiple synchronous processes in a social interaction setting (i.e., watching a TV show with another person) indicating that an experience is not merely experienced together but shared together. Each of these processes appears to provide unique contributions that give rise to a shared experience and that jointly promote the development of social connections between individuals. Our work demonstrates that these processes can be observed, measured, and analyzed together in a naturalistic setting, which provides an important advance for developing a more unified framework for studying social interactions and relationships.

## Methods

### Participants

We recruited 86 participants who either watched the four episodes of *Friday Night Lights* alone (*N* = 22, 68% female; M_age_ = 18.90, SD = 0.92) or as a dyad with another participant (*N* = 64, 32 dyads; 68% female; *M*_*age*_ = 19.26, SD = 1.13). Five dyads recognized each other, and two dyads identified themselves as close friends. Omitting the dyads that recognized each other did not affect the main results. Twelve of the dyads were mixed-gender pairs. Two dyads did not return for the second session and were excluded from analyses. All participants provided informed consent to participate in this study and whether they agreed to sharing identifiable images. Participants received $10 per hour or course credit in compensation for their participation. This study was approved by the Institutional Review Board at Dartmouth College.

We also recruited participants from the Amazon Mechanical Turk workplace (*N* = 192, 55% female, M_age_ = 37.11, SD = 10.71) to periodically self-report emotion ratings while watching the first episode of *Friday Night Lights* online. Four subjects were excluded who did not respond to more than 75% of the time when they were asked to report how they were feeling. All participants provided informed consent to participate in this study and received monetary compensation ($9). This study was approved by the Institutional Review Board at Dartmouth College and is described in more detail in Chang et al.^9^.

### Procedure and materials

Participants watched four episodes of a TV character drama (*Friday Night Lights*) in two separate sessions scheduled an average of 2.65 (SD = 2.78) days apart. For dyad participants, we did not explicitly preclude them from meeting each other between sessions which would have been both unenforceable and unrealistic in a campus setting. However, there was no significant difference t(27) = 1.4, *p* = 0.10 in the increase of connection ratings from between sessions, M = 0.45, SD = 0.52, compared to the within-session increase in connection ratings M = 0.22, SD = 0.38. No participants reported viewing the show prior to the experiment. In the shared viewing condition, both participants were briefly introduced to one another as they filled out consent and demographics forms to check if they were acquainted with one another, which they indicated on a separate form. The two participants sat side-by-side at a slight angle towards the monitor and were instructed to watch the shows naturally as they would with a friend. Participants in the solo viewing paradigm watched alone in the same room. All participants wore a head-mounted GoPro camera^61^.

The episodes were presented on a 15-inch Apple Macbook Pro laptop and the average length of each episode was 43.6 min (*range* = 42–46 min). Facial behaviors were recorded at a resolution of 1280 × 720 resolution at 120hz using a GoPro Hero4 Black camera attached to the custom head-mount setup^61^. Electrodermal activity was also recorded during the sessions at 500 Hz using the Acqknowledge software with a BIOPAC MP150 system. EL507 electrodes treated with GEL101 0.5%-NaCL electrode paste were attached to the medial phalanges of the third and fourth fingers on the left hand.

After each episode, participants rated 13 characters on eight social impression dimensions and three character relationship dimensions (i.e., How much does character A like, trust, or listen to character B?; not discussed in this paper) on a 0–100 scale in separate rooms (see Table 1 for exact wording). In addition to the character ratings, dyad participants reported on a Likert scale on how much they enjoyed the show (1: Not enjoyed, to 9: Very enjoyed) and how much they felt connected to the other person (1: Not connected, to 9: Very connected)^3,50^.

**Table 1:** Questions asked to participants.

*Character impression questions*
How *annoying* do you find this character?
How much do you *like* this character?
How *attractive* do you find this character?
How much do you want to be *friends* with this character?
How much do you want to *be* this character?
How much does this character *remind* you of someone you know?
How much do you *relate* to this character?
How much do you *care* what happens to this character?
*Enjoyment question*
How much did you enjoy the show?
*Social connection question*
How much do you feel connected to the other participant?

For online participants, we delivered the first episode of *Friday Night Lights* through a web application built using Flask (http://flask.pocoo.org/), jsPsych (https://www.jspsych.org/), psiTurk (https://psiturk.org/), and MySQL (https://www.mysql.com/), served on an NGNIX (https://www.nginx.com/) webserver hosted in our laboratory (https://github.com/cosanlab/moth_app, https://github.com/cosanlab/moth_turkframe). The video was paused at pseudo-random intervals at approximately 200 to 280 s at which participants rated the intensity of 16 emotions they were experiencing including joy, surprise, sadness, fear, anger, disgust, contempt, relief, envy, shame, interest, elation, satisfaction, guilt, hope, and pride. Each subject provided on average 11.46 ratings (SD = 1.94), totaling 66,714 ratings across the six emotion categories of primary interest (joy, surprise, sadness, fear, anger, and disgust). These emotion ratings were averaged across subjects, interpolated, and smoothed with a 30-s sliding window. See ref. ^9^ for additional details.

### Video preprocessing and feature extraction

Each face recording video was aligned and trimmed to the stimulus video by finding the audio offset that maximizes the correlation of the audio envelope using the FaceSync toolbox^61^ which yields comparable results (within 3 ms error) to the current gold standard of manual alignment. Facial features including 20 action units and 6 emotion predictions were extracted using the FACET algorithm accessed via iMotions 6.0^66^ software. FACET is a deep convolutional neural network that improves upon the computer expression recognition toolbox (CERT)^62^ and predicts evidence of different emotions and action units based on the FACS annotations^69,95^ coded by experts using pixel information from each frame of the video. Predictions values are represented as evidence scores which are logarithmic odds of the presence of emotional facial expressions. The extracted facial feature predictions were downsampled to 1hz. We applied a minimum face detection failure cutoff so that videos in which the algorithm failed to detect a face for more than 10% of the video were excluded from the analysis resulting in the exclusion of two dyads. In the solo viewing condition, one subject was excluded due to file corruption while transferring video data.

### Measuring facial expression synchrony in long and short timescales

For both positive (i.e., joy facial expression) and negative valence (i.e., max of anger, fear, disgust, sadness, and contempt^66^,) evidence time series, we computed the pairwise intersubject similarity using a Pearson correlation over the duration of each episode. This synchrony measure was computed for every pairwise combination of subjects in the alone group resulting in 210 correlations, (*n* = 21),

1 $$\frac{n\,* \,(n-1)}{2}$$

and for the pairs of participants who physically participated together in the dyad group resulting in 28 correlations (*n* = 56). Pseudo pairs in dyads resulted in 1512 correlations for each episode

2 $$\frac{n\,* \,(n-1)}{2}-28$$

The synchrony metric used here captures the degree to which facial expressions covary at similar moments in time over the course of the episode. The significance of the average level of synchrony was determined by a nonparametric subject-wise bootstrapping procedure in which subjects were resampled with replacement over 5000 iterations to account for subject-level exchangeability and independence^64^. Similarly, the between-group comparison was computed using a subject-wise permutation technique that generates a null distribution of the correlation matrix by shuffling the participants between the groups repeatedly for 5000 iterations^64,96^. We also computed hypothesis tests by generating a null distribution of circularly shifted time-series data, which preserves the overall autoregressive and temporal structure of the data^63,65^. To check for potential time-lagged coupling of expressions between dyads, we computed the cross-correlation between 3 s for each dyad at 100 ms intervals (Supplementary Fig. S4) and found that the majority (>50%) of the maximum synchrony occurred within 1 s (median offset = 850 ms) of each other suggesting a rapid synchronous response to external stimuli.

To measure moment-to-moment synchrony, a 30-s moving window correlation was computed for all pairwise combinations of subjects for the alone group and for each pair of dyads who watched together for the positive facial expression synchrony. These correlation values were z-transformed and compared between groups to generate a t-statistic at every second. The moments when the group differed significantly were computed using a cluster-based nonparametric test^67^ to detect consecutive moments showing group differences. After identifying the moments in the episode at which dyads showed greater synchrony of positive facial expressions, we compared which emotions were dominant at those times. We used average emotion ratings collected from 192 participants from Amazon Mechanical Turk who viewed the first episode of the show which was periodically paused to allow them to rate their emotions (see Supplementary Methods). The emotion ratings were averaged across subjects, interpolated, and smoothed using a sliding window of 30 s. These ratings were then correlated with the moment-to-moment synchrony of joy facial expressions. In addition, we computed the average rating for each cluster and contrasted the difference between the joy and the average of other emotion ratings (Supplementary Fig. S3).

### Synchrony of facial expressions in predicting social connectedness

The degree of social connection that each dyad participant reported towards their partner after every episode was averaged to represent the combined affiliation level following the shared viewing of each episode. These values were then compared to the positive and negative facial expression synchrony levels for each episode using a Spearman rank correlation to assess the monotonic relationship as unit increases in synchrony may not necessarily reflect a proportional change in connectedness. We also tested for a linear relationship in conjunction with time effects using a linear mixed-effects regression (M1) with random effects at the dyad level estimating the average connection ratings (Conn) from the positive facial expression synchrony (PosExpSync), episode number as an indicator of time (Epn), and their interactions. This model is written out using the R, lmer4 syntax^97^.

3 $$M1:{Conn}=\, 	b0+b1* {PosExpSync}+b2* {PosExpSync}* {Epn} \\ 	 +b3* {Epn}+(1{{{{{\rm{|}}}}}}{Dyad})$$

Prediction of connectedness ratings was computed using the pairwise synchrony for each of the six emotion predictions of joy, anger, surprise, fear, sadness, and disgust. We evaluated our model using a leave-one-dyad-out cross-validation scheme where every fold estimated a new model using a penalized L2 regression with a nested 5-fold cross-validation search for the optimal regularization strength for values of [0.1, 1.0, 10.0]. The accuracy of the overall model was evaluated as the Pearson correlation between actual average connectedness ratings and the predicted connectedness ratings across all folds.

To evaluate the significance of the contributions from each emotion synchrony, we fit a linear mixed-effects regression (M2) with random dyad effects to predict connection (Conn) based on the synchronization of the six canonical emotional expressions.

4 $$M2:{Conn}= \, 	b0+b1* {Joy}+b2* {Anger}\\ 	 +b3* {Surprise}+b4* {Fear}+ b5* {Sad} \\ 	 +b6* {Disgust}+{Epn} +(1{{{{{\rm{|}}}}}}{Dyad})$$

### Latent shared response model estimation

The latent shared response model was fit using the SRM module^68^ in the BrainIAK package (Brain Imaging Analysis Kit, http://brainiak.org). The latent shared response performs a reduced-rank factorization of the multivariate facial expression data into *k* shared response features with an orthogonal transformation matrix for each subject. This allows each participant’s time by action unit data to be decomposed into a subject-specific action unit by shared response transformation matrix and a common time by shared response matrix. This procedure is akin to estimating a joint PCA, where each participant has a unique transformation into the common latent space shared across participants. The number of shared responses *k* was determined by iterating through the full range of *k’s* that maximizes the shared response similarity (i.e., time) for all participants from both groups (i.e., dyad and alone) across the four episodes resulting in *k* = 2 components (Supplementary Fig. S6). To interpret the shared response trajectories estimated for the first episode, we correlated each trajectory with the joy, anger, surprise, fear, sadness, and disgust crowdsourced emotion ratings both with and without detrending which yielded identical results (Supplementary Table S3).

The spatial configuration similarity of facial expressions was computed for each shared response by taking the idiosyncratic transformation matrix of each subject for each feature and computing the intersubject similarity for each group. The intersubject similarities in the spatial configuration of facial expression were then compared with connection ratings using Spearman’s rank correlation. The average weights and subject-specific facial expression maps for each feature were plotted using the Python Facial Expression Analysis Toolbox (Py-FEAT; ref. ^98^) and were depicted by scaling using cubic exponentiation for clarity (see ref. ^9^ for more details about visualization methods). This software allows the unique facial expression of each participant to be displayed without revealing their individual identity.

### Electrodermal activity preprocessing

Electrodermal activity data was bandpass filtered with a lower bound of 0.005 Hz and an upper bound of 5 Hz^99^ and subsequently downsampled to 1 Hz. Three dyads who did not show signs of electrodermal activity due to either participants being non-responders or acquisition error were removed from analyses. EDA synchrony was measured as the Pearson correlation between the log-transformed EDA time series data of dyads. The degree of EDA synchrony (EDASync) was compared to the average connection ratings (Conn) for each dyad using a Spearman rank correlation for each episode, and the overall effect was evaluated using a linear mixed-effects regression as shown in M3.

5 $$M3:{Conn}=\,	 b0+b1* {EDASync}+b2* {EDASync}* {Epn} \\ 	 +b3* {Epn}+(1{{{{{\rm{|}}}}}}{Dyad})$$

The global and dynamic moment-to-moment EDA synchronies were calculated and statistically tested for significance in the same manner described above for the facial expression synchrony.

### Impression similarity analysis

After each episode, participants answered eight questions for each of the 13 characters (Table 1). We conducted a principal components analysis to find orthogonal dimensions of the impression rating space. Character ratings were demeaned within each subject to adjust for individual variability and we treated each character rating from each subject as samples of the impression space captured with the eight questions. We found that at least 5 components were necessary to retain approximately 90% of the variance. We projected the impression ratings for each character for each subject into this space and computed the intersubject similarity^10,11^ separately for each character and then averaged across characters. We then fit a linear mixed-effects model (M4) in which impression similarity (ImpSim), episode number (Epn), and their interaction were used to estimate average connection ratings.

6 $$M4:{Conn}=\,	 b0+b1* {ImpSim}+b2* {ImpSim}* {Epn}\\ 	 +b3* {Epn}+(1{{{{{\rm{|}}}}}}{Dyad})$$

### Structural equation modeling

We fit our structural equation model predicting connection ratings with shared experience as a latent factor using the lavaan package in *R*^100^. Shared experience latent factor was estimated using measurements of positive temporal facial expression synchrony, positive spatial facial expression synchrony, EDA synchrony, and character impression similarity. We modeled residual correlations between the temporal and spatial facial expression synchrony measures to account for the fact that they are both derived from facial expression data. We also included residual correlations between episode number and the other synchrony measurements to control for the residual covariance due to time.

### Softwares and packages used in analyses

We used the following tools for statistical analyses and visualizations: Pandas^101^, brainiak^68^, seaborn^102^, matplotlib^103^, scipy^104^, numpy^105^, sklearn^106^, py-feat^98^, FaceSync^61^, nltools^107^ in Python, and lme4^108^, lmerTest^109^, and lavaan^100^ in R.

### Reporting summary

Further information on research design is available in the Nature Portfolio Reporting Summary linked to this article.

### Supplementary information

Supplementary Information

Reporting Summary

## Data Availability

Data are available at https://osf.io/6ejvg/.

## Code availability

All code to reproduce the analyses and figures is available on https://github.com/cosanlab/FNL_dyad^110^.
