# Supplementary material for: Synchronized affect in shared experiences strengthens social connection
Source: Commun Biol. 2023 Oct 28;6:1099. doi: 10.1038/s42003-023-05461-2 (PMC10613250; doi:10.1038/s42003-023-05461-2)
Supplement: Supplementary file 1 — Supplementary Information [file 42003_2023_5461_MOESM1_ESM.pdf]

Supplementary information

Synchronized affect in shared experiences strengthens social connection

Jin Hyun Cheong*, Zainab Molani, Sushmita Sadhukha, and Luke J. Chang*

* author for correspondence

Supplementary Figure 1
Supplementary Figure 2
Supplementary Figure 3
Supplementary Figure 4
Supplementary Figure 5
Supplementary Figure 6
Supplementary Table 1
Supplementary Table 2
Supplementary Table 3

Supplementary Figures

Supplementary Figure 1. Temporal synchrony of facial expressions and EDA across conditions

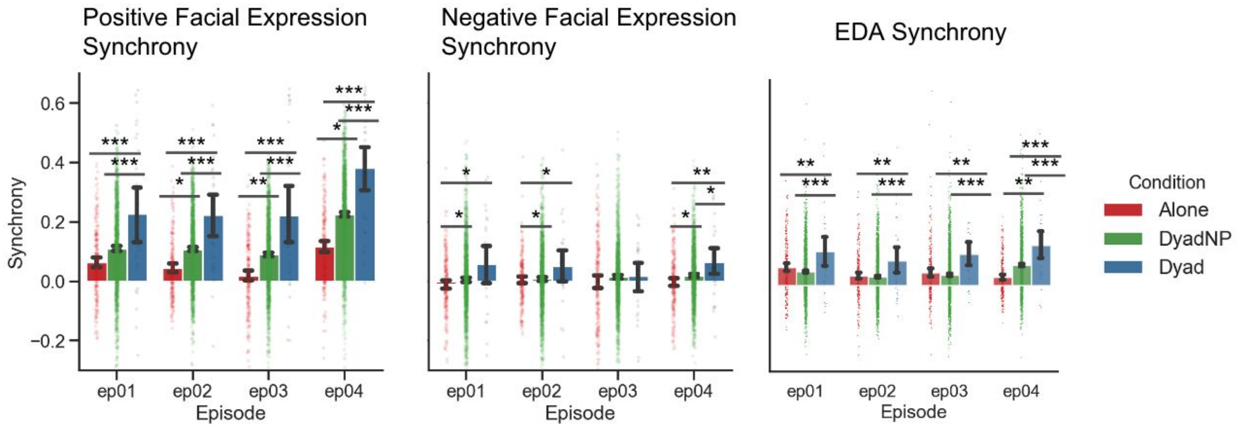

Supplementary Figure 1. Comparison of temporal facial expression and EDA synchrony between all pairwise combinations of participants who watched alone (Alone), pairs of participants who watched in dyads (Dyad), and all pairwise combinations of participants in the dyad group less those who actually watched together (non-pairs, DyadNP).

Supplementary Figure 2. Moment-to-moment synchrony comparison between groups

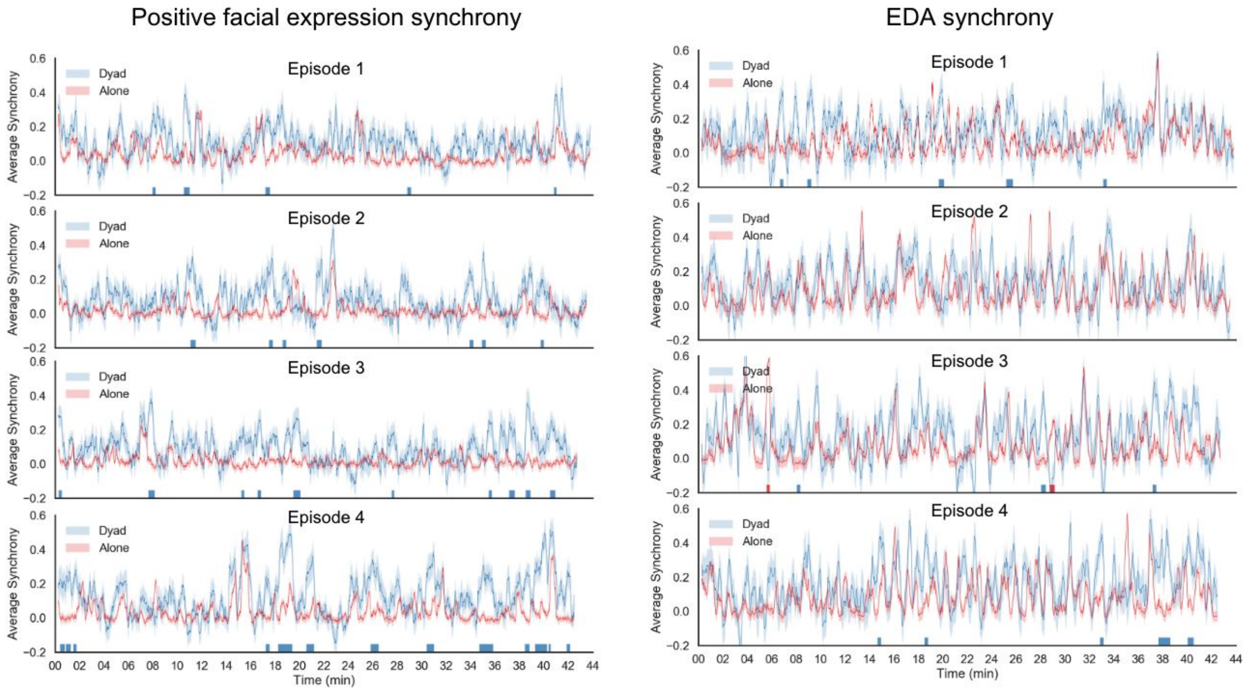

Supplementary Figure 2. Moment-to-moment dynamic synchrony of positive facial expressions (left) and EDA (right) comparison between dyad and alone groups across all episodes.

Supplementary Figure 3. Average crowdsourced emotion ratings at scenes with greater dyadic synchronization in episode 1.

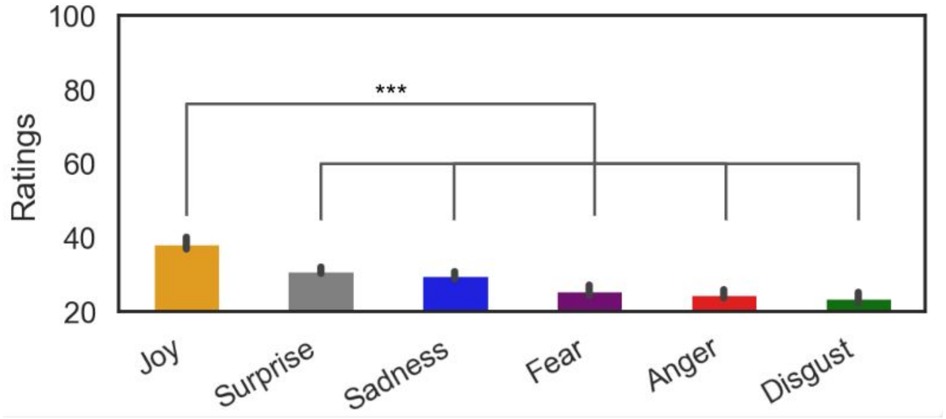

Supplementary Figure 3. Average crowdsourced emotion ratings at scenes with greater dyadic synchronization in episode 1. Joy emotion ratings were compared with the average across all other ratings. Average joy rating across clusters in which dyads showed significantly higher synchrony than the alone group was greater than the ratings of other emotions $\beta = 12.14$, $t(24) = 4.08$, $p < .001$.

Supplementary Figure 4. Distribution of crowdsourced emotion ratings for episode 1.

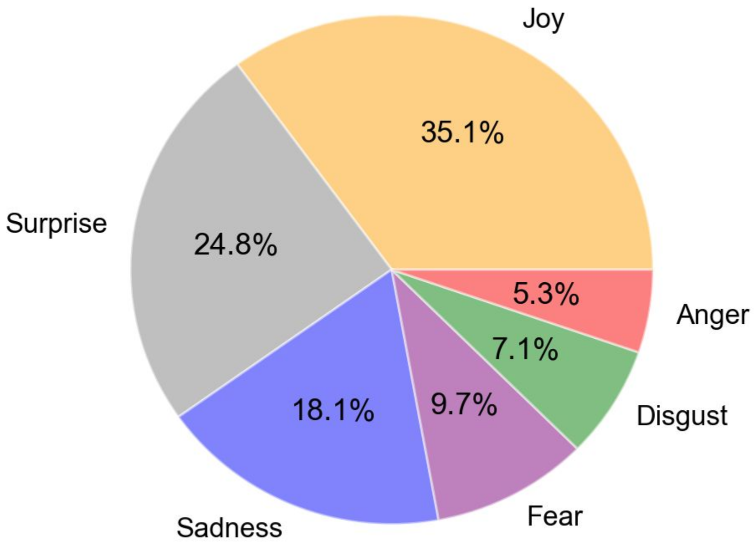

Supplementary Figure 4. Distribution of crowdsourced emotion ratings for episode 1. The pie chart illustrates the percentage of the show at which each emotion was rated the strongest elicited emotion from the average self-reported ratings collected from online participants.

Supplementary Figure 5. Offset maximizing synchrony in dyads.

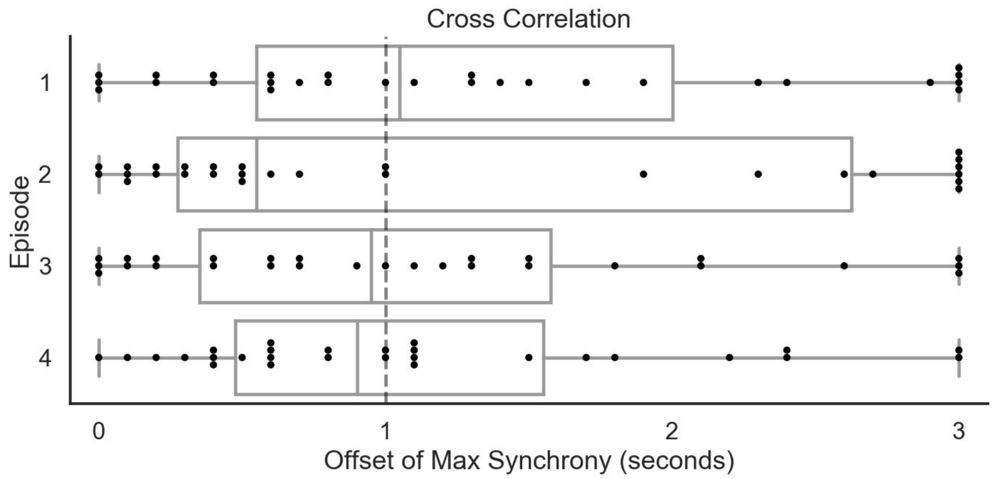

Supplementary Figure 5. Distribution of offsets that maximizes the cross correlation for each dyad on positive facial expressions. Each dot represents a dyad in each episode where the cross correlation of their facial expression synchrony in 10 Hz was the highest searched within ±3 seconds. The distribution of offsets indicates that the majority of subjects synchronized within a second of each other with a median offset of 850 ms. The dotted line marks the offset of 1 second.

Supplementary Figure 6. Average Intersubject Correlation of Shared Responses

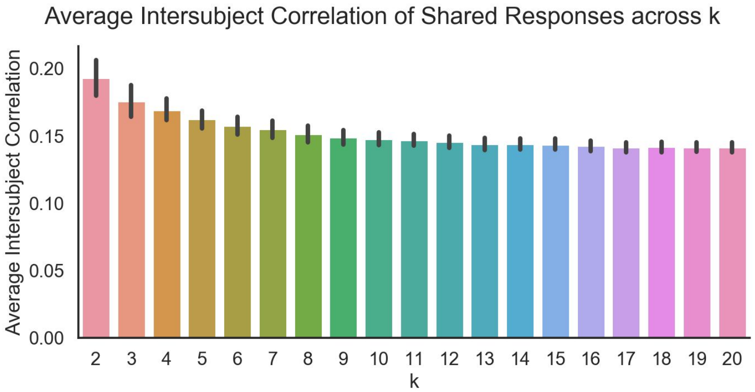

Supplementary Figure 6. Average Intersubject Correlation of Shared Responses. The average intersubject correlation across the shared response estimated for k=[2, 3, …, 20] are shown. Error bars indicate 95% bootstrapped confidence intervals.

Supplementary Tables

Supplementary Table 1. Average group correlations for positive and negative facial expressions synchrony and EDA synchrony

Episode	*Group*	*Positive Expression Synchrony*	*Negative Expression Synchrony*	*EDA Synchrony*
		Average r (subject-wise bootstrapped significance, circle shift randomization testing)		
1	dyad<br>alone	.23 (***, ***)<br>.06 (***, ***)	.06 (*n.s.*, *)<br>-.01 (*n.s.*, *n.s.*)	.11 (**, ***)<br>.06 (***, ***)
2	dyad<br>alone	.22 (***, ***)<br>.04 (**, ***)	.05 (*n.s.*, *)<br>.00 (*n.s.*, *n.s.*)	.08 (*, ***)<br>.03 (**, ***)
3	dyad<br>alone	.22 (***, ***)<br>.02 (*n.s.*, *)	.02 (*n.s.*, *n.s.*)<br>.00 (*n.s.*, *n.s.*)	.10 (*, ***)<br>.04 (***, ***)
4	dyad<br>alone	.38 (***, ***)<br>.12 (***, ***)	.07 (*n.s.*, ***)<br>.00 (*n.s.*, *n.s.*)	.13 (***, ***)<br>.03 (**, ***)

n.s.: not significant, * p<.05, ** p<.01, ***, p<.001

Supplementary Table 2. Structural equation modeling fit results.

Parameter Estimate	*Unstandardized*	*Standardized*	*p*
Latent variables			
Shared Exp → Temporal Sync	1.00	.92	.000
Shared Exp → Spatial Sim	.98 (.19)	.59	.000
Shared Exp → EDA Sync	.30 (.07)	.57	.000
Shared Exp → Impression Sim	.22 (.08)	.28	.009
Error in Temporal Sync	.01 (.01)	.16	.287
Error in Spatial Sim	.08 (.02)	.65	.000
Error in EDA Sync	.01 (.00)	.67	.000
Error in Impression Sim	.03 (.00)	.92	.000
Covariance of Temporal Sync - Spatial Sync	-.01	-.29	.401
Covariance of Episode Num - Temporal	(.01)	.57	.018
Sync	.06 (.03)	.37	.002
Covariance of Episode Num - Spatial Sync	.12 (.04)	.08	.465
Covariance of Episode Num - EDA Sync	.01 (.01)		
Covariance of Episode Num - Impression		.29	.004
Sim	.05 (.02)		
Structural Model			
Shared Exp → Connection Ratings	4.41 (.94)	.59	.000
Episode Num → Connection Ratings	.35 (.13)	.24	.001
Error in Connection Ratings	1.61 (.27)	.60	.000

Note: $\chi^2(4) = 6.42$, p =.170; GFI = .98; NFI = 96; CFI = .98; RMSEA [95%CI] = .07 [.00, .17] , SRMR = .042.

Supplementary Table 2. Unstandardized, standardized, and significance levels for the structural equation model (Standard error in parentheses; N = 110).

Supplementary Table 3. Average shared response trajectories correlated with crowdsourced self-reported emotion ratings.

Shared Response	Joy	Disgust	Anger	Surprise	Sadness	Fear
1	**.42 *** (0.12***)**	0.01 (-0.02)	-.18 *** (-0.12***)	-.26 *** (-0.27***)	-.31 *** (-0.41 ***)	-.40 *** (-0.24***)
2	-.38 *** (0.10***)	.11 *** (0.03)	-.16 *** (0.30***)	.09 *** (0.63***)	0.23 *** (0.49***)	**.29 *** (0.65***)**

Note: Correlations are shown for with detrending and without detrending in parentheses. Emotions with the highest correlations for each shared response are in bold. *p<.05, **p<.01, ***p<.001
