# Supplementary material for: Synchronized affect in shared experiences strengthens social connection
Source: Commun Biol. 2023 Oct 28;6:1099. doi: 10.1038/s42003-023-05461-2 (PMC10613250; doi:10.1038/s42003-023-05461-2)
Supplement: Supplementary file 2 — Reporting Summary [file 42003_2023_5461_MOESM2_ESM.pdf]

nature research

Corresponding author(s): Luke J. Chang

Last updated by author(s): May 21, 2023

Reporting Summary

Nature Research wishes to improve the reproducibility of the work that we publish. This form provides structure for consistency and transparency in reporting. For further information on Nature Research policies, see Authors & Referees and the Editorial Policy Checklist .

Statistics

For all statistical analyses, confirm that the following items are present in the figure legend, table legend, main text, or Methods section.

n/a	Confirmed	
☐	☒	The exact sample size (n) for each experimental group/condition, given as a discrete number and unit of measurement
☐	☒	A statement on whether measurements were taken from distinct samples or whether the same sample was measured repeatedly
☐	☒	The statistical test(s) used AND whether they are one- or two-sided<br>*Only common tests should be described solely by name; describe more complex techniques in the Methods section.*
☐	☒	A description of all covariates tested
☐	☒	A description of any assumptions or corrections, such as tests of normality and adjustment for multiple comparisons
☐	☒	A full description of the statistical parameters including central tendency (e.g. means) or other basic estimates (e.g. regression coefficient) AND variation (e.g. standard deviation) or associated estimates of uncertainty (e.g. confidence intervals)
☐	☒	For null hypothesis testing, the test statistic (e.g. F, t, r) with confidence intervals, effect sizes, degrees of freedom and P value noted<br>*Give P values as exact values whenever suitable.*
☒	☐	For Bayesian analysis, information on the choice of priors and Markov chain Monte Carlo settings
☒	☐	For hierarchical and complex designs, identification of the appropriate level for tests and full reporting of outcomes
☐	☒	Estimates of effect sizes (e.g. Cohen's d, Pearson's r), indicating how they were calculated

Our web collection on statistics for biologists contains articles on many of the points above.

Software and code

Policy information about availability of computer code

Data collection	Facial behaviors were recorded using GoPro HERO 4 cameras. Character ratings were collected through custom code written in Meteor 1.9.0 hosted on https://cosanlabresearch.dartmouth.edu.
Data analysis	Python 3.6 was used to analyze the data with packages Pandas, Numpy, Seaborn, Matplotlib, Scikit-learn, Scipy, Nltools, brainiak, FEAT, and FaceSync. We also used R lme4, lmerTest, and lavaan packages to analyze data. Facial behavior features were extracted using the FACET algorithm accessed through iMotions Biometric Research Platform 6.0. Python Facial Expression Analysis Toolbox (FEAT; V0.0.1) to visualize the facial features.

For manuscripts utilizing custom algorithms or software that are central to the research but not yet described in published literature, software must be made available to editors/reviewers. We strongly encourage code deposition in a community repository (e.g. GitHub). See the Nature Research guidelines for submitting code & software for further information.

Data

Policy information about availability of data

All manuscripts must include a data availability statement. This statement should provide the following information, where applicable:

- Accession codes, unique identifiers, or web links for publicly available datasets
- A list of figures that have associated raw data
- A description of any restrictions on data availability

Data is available at https://osf.io/6ejvg/ and code is available on https://github.com/cosanlab/FNL_dyad

Field-specific reporting

Please select the one below that is the best fit for your research. If you are not sure, read the appropriate sections before making your selection.

- [ ] Life sciences
- [x] Behavioural & social sciences
- [ ] Ecological, evolutionary & environmental sciences

For a reference copy of the document with all sections, see nature.com/documents/nr-reporting-summary-flat.pdf

Behavioural & social sciences study design

All studies must disclose on these points even when the disclosure is negative.

Study description	This is a quantitative experimental study.
Research sample	Participants are undergraduate students of Dartmouth College, N=86 of which N=64 watched in dyads (32 dyads; 68% female; Mage = 19.26, SD= 1.13) and N=22 watched alone (68% female; Mage = 18.90, SD =.92). A separate group of participants was recruited online across the United States to collect self-report emotion ratings (N = 192, 55% female, Mage = 37.11, SD = 10.71).
Sampling strategy	Participants were conveniently recruited from the Dartmouth College subject pool for either pay or for course credit. We calculated our total target sample of about 80 with approximately 30 dyads that is comparable to many dyadic correlation studies investigating facial expressions (Fridlund 1991; Wagner and Smith 1991; Hess et al. 1995; Jakobs et al. 2001; Konvalinka et al. 2011; Riehle et al. 2017).
Data collection	Data were collected with Macbook Pro computers, GoPro cameras, and pen and paper. The experimenter was not present in the room during the experiment and was blind to the hypothesis of the study.
Timing	Data were collected from September 2016 through October 2017 for participants recruited at Dartmouth College. Online data collection was done through February and March 2018.
Data exclusions	Two dyads were excluded as they did not return for the second viewing session due to scheduling conflicts. Additionally, two dyads were excluded whose videos resulted in failed face detection at more than 10% throughout the sessions. Lastly, data from 1 participant who watched alone could not be analyzed due to video file corruption while transferring videos.
Non-participation	No participants dropped out other than the two mentioned in exclusions.
Randomization	Participants signed up to be in either study group. Our main effects are within-group effects.

Reporting for specific materials, systems and methods

We require information from authors about some types of materials, experimental systems and methods used in many studies. Here, indicate whether each material, system or method listed is relevant to your study. If you are not sure if a list item applies to your research, read the appropriate section before selecting a response.

Materials & experimental systems

n/a	Involved in the study	
x		Antibodies
x		Eukaryotic cell lines
x		Palaeontology
x		Animals and other organisms
x		Human research participants
x		Clinical data

Methods

n/a	Involved in the study	
x		ChIP-seq
x		Flow cytometry
x		MRI-based neuroimaging
